# Parenting-by-gender interactions in child psychopathology: attempting to address inconsistencies with a Canadian national database

**DOI:** 10.1186/1753-2000-4-5

**Published:** 2010-01-27

**Authors:** Dillon T Browne, Adefowope Odueyungbo, Lehana Thabane, Carolyn Byrne, Lindsay A Smart

**Affiliations:** 1 Department of Psychology, University of Guelph, Guelph, Canada; 2 Department of Clinical Epidemiology and Biostatistics, McMaster University, Hamilton, Canada; 3 Centre for Evaluation of Medicines, St Joseph's Healthcare Hamilton - a Division of St Joseph's Health System, Hamilton, Canada; 4 Faculty of Health Sciences, University of Ontario Institute of Technology, Oshawa, Canada

## Abstract

**Background:**

Research has shown strong links between parenting and child psychopathology. The moderating role of child gender is of particular interest, due to gender differences in socialization history and in the prevalence of psychiatric disorders. Currently there is little agreement on how gender moderates the relationship between parenting and child psychopathology. This study attempts to address this lack of consensus by drawing upon two theories (self-salience vs. gender stereotyped misbehaviour) to determine how child gender moderates the role of parenting, if at all.

**Methods:**

Using generalized estimating equations (GEE) associations between three parenting dimensions (hostile-ineffective parenting, parental consistency, and positive interaction) were examined in relationship to child externalizing (physical aggression, indirect aggression, and hyperactivity-inattention) and internalizing (emotional disorder-anxiety) dimensions of psychopathology. A sample 4 and 5 year olds from the National Longitudinal Survey of Children and Youth (NLSCY) were selected for analysis and followed over 6 years (N = 1214). Two models with main effects (Model 1) and main effects plus interactions (Model 2) were tested.

**Results:**

No child gender-by-parenting interactions were observed for child physical aggression and indirect aggression. The association between hostile-ineffective parenting and child hyperactivity was stronger for girls, though this effect did not reach conventional levels of statistical significance (*p *= .059). The associations between parenting and child emotional disorder did vary as a function of gender, where influences of parental consistency and positive interaction were stronger for boys.

**Discussion:**

Despite the presence of a few significant interaction effects, hypotheses were not supported for either theory (i.e. self-salience or gender stereotyped misbehaviour). We believe that the inconsistencies in the literature regarding child gender-by-parenting interactions is due to the reliance on gender as an indicator of a different variable which is intended to explain the interactions. This may be problematic because there is likely within-gender and between-sample variability in such constructs. Future research should consider measuring and modelling variables that are assumed to explain such interactions when conducting gender-by-parenting research.

## 

There exists a great deal of psychosocial literature that examines the associations between parenting practices and psychological development in children. A need persists for high quality longitudinal research that uncovers the precise nature of these effects, namely, the complex relationships between early predictor variables and later psychological outcomes [1,2]. Also, scientists must continue to recognize that negative parent-child environments are not equally harmful to all children [3]. There may be constitutional factors or other environmental situations that modify certain aspects of risk experience [4-6]. Based on child gender differences in socialization and psychopathology, it is possible that gender will moderate the relationship between parenting and child psychopathological outcomes [5].

Developmental Systems Theory suggests that child development is attributable to "dynamic person-context relationships" that are characterized by organizational complexity across multiple levels of analysis [7,8]. Person by context interactions are critical, where individual differences can moderate expected outcomes in response to ecological settings and vice versa. Thus, parenting behaviours that are analyzed individually, without accounting for child variables, may lead to "biased or misleading" results [p. 41; [9]] because they assume the observed relationships are operative for all children. Researchers should investigate the specific associations between parenting and child externalizing and internalizing disorders, where interactions amongst predictors and child variables are sought, so that our understanding of psychopathological development is accurate and capable of appropriately informing future research, clinical practice and public policy.

### Parenting constructs as risk factors

Developmental science may be moving beyond main effect interpretations parental risks, though a foundation in the basics of risk factor research is necessary. A risk factor is a biological or psychosocial danger that increases one's propensity to experience a negative outcome [10]. There are three major classifications of environmental risk experience for childhood psychopathology, all of which have implications on (though are not exclusive to) the parent-child relationship. These include a) deficiencies in stable positive relationships, b) deficiencies in solidarity and cohesion within the family and other social systems, and c) deficiencies in interpersonal stimulation [3]. Risks associated with parenting are proximal risk factors, or are *directly *involved in the development of child behavioural and emotional disorders [2,3].

Parenting research has benefited from studies that examine child outcomes associated with parenting dimensions of hostile-ineffective parenting, parental consistency, and positive parent-child interactions, all of which are assessed by Strayhorn and Weidman's Parenting Practices Scale [11]. For example, Miller, Jenkins and Keating [12] found that experiencing parental hostility greatly increased the odds of a child exhibiting a behaviour disorder. Low parental consistency was also associated with higher odds of a disorder, though the effect was less extreme. Moreover, the authors determined that these parenting constructs operate independently of the relationship between socioeconomic status and behavioural disorders in children. Certainly, a plethora of literature has illustrated the importance of effectiveness of disciplinary strategies, regularity, and warmth during interaction on the development of physical and indirect aggression [13-16], hyperactivity-inattention [12,17,18] and emotional disorder-anxiety in children [19-22]. These parenting dimensions are often the foci of parent-training interventions and are strongly associated with child behaviour variables. It should be noted that the parenting dimensions of concern in this study are not the only influential domains of parent child-relationships; attachment patterns, proximity seeking, protective care giving, empathy, shared attention and turn-taking are some other parental constructs that influence development across childhood [5].

### The moderating effect of gender

Before discussing the moderating role of gender, differences in the prevalence of psychopathology across boys and girls must be acknowledged. Literature has shown that boys show higher rates of externalizing disorder, while females exhibit higher rates of internalizing disorder [23-25]. A nationwide prevalence study of 1400 American children reported that boys exhibit higher levels of ADHD, conduct disorder and oppositional defiant disorder [26]. A similar Canadian study of 21,455 children reported higher rates of behaviour problems in boys and higher rates of emotional maladjustment in girls, though the latter effect was not observed until age four and there were some developmental variations [27]. Some investigations from non-Western settings have failed to report such differences [28].

Research has acknowledged that child characteristics, such as temperament and developmental status, can moderate the impact of familial risk factors for child psychopathology [4,6]. Gender may also influence children's responses to environmental experience, such as parental disciplinary practices [6,29]. Previous research has found that parenting practices predict externalizing behaviour for boys [30] and internalizing behaviour for girls [31]. However, other research suggests that this relationship is more nuanced. Kim and colleagues [29] examined the differential effects of parental hostility and inconsistency on gender-typed stereotypical misbehaviour in preschoolers. The authors defined stereotype congruency as the degree to which children's behaviours were consistent with social expectations and norms for children's behavioural and emotional conduct. That is, submissiveness and emotional dependence are generally more socially acceptable when exhibited by girls, while gross motor activity, physical aggression, and rough-and-tumble play are more acceptable for boys [29]. The study found internalizing disorders in girls and externalizing disorders in boys (i.e. stereotype congruent misbehaviour) to be associated with permissiveness. Externalizing behaviour in girls and internalizing disorders in boys (i.e. stereotype incongruent misbehaviour) was associated with parental hostility. Other research has found that parental consistency and monitoring were only important predictors of externalizing symptoms for male adolescents [32].

Research concerning the differential associations between parenting and child psychopathology must also account for gender differences in the types of externalizing symptoms. For example, girls are more likely to express externalizing behaviour through indirect aggression [IA; [33]]. Also called relational aggression, this behaviour causes harm through attacks on an individual's relationships and feelings of social inclusion. Many studies illustrate the greater prevalence of IA in girls, linking this phenomenon with environmental factors [34]. However, some research has shown that boys are more susceptible when predicting IA. For example, using sophisticated modelling techniques, Vaillancourt and colleagues [35] reported that parental consistency and positive interaction are significant predictors of membership in an "increasing use of indirect aggression" trajectory for boys only. A similar study by the same research group predicted the development and change of combined indirect and physical aggression. Their findings illustrated the importance of gender and hostile-ineffective parenting main effects in predicting trajectories of aggression across time, though parenting-by-gender interactions were not a focus of this study [36].

### Two competing theoretical frameworks

Uncovering significant interactions between parenting predictor variables and child characteristics will help provide the most accurate information regarding when, where and how the environmental effects of family-based risk factors truly operate. Caron and colleagues [9] have noted that there is a paucity of literature illustrating the "specificity of effects" (p. 35) between parenting dimensions and child psychopathology, where specificity refers to unique, differential, interactive, and moderator effects. The interactive effects of gender are often not present or explicit. Indeed, Crick and Zahn-Waxler [33] suggest that a great deal of research examining the development of child psychopathology has ignored the influence of child gender. They note that studies which do examine the role of gender in the development of psychopathology focus on main effect interpretations, ignoring the interactions between gender and other important predictor variables.

Currently, there exists no consensus on exactly *how *child gender moderates the relationship between parenting and child psychopathology. Two theories are utilized to guide the present investigation: 1) Kim, Arnold, Fisher and Zeljo's [29] findings which we call the *theory of gender stereotyped misbehaviour *and 2) Rosenfield, Lennon White and Raskin's [37]*theory of self-salience and psychopathology*, which is being adapted to address the parenting-by-gender issue. Kim and colleagues [29] findings suggest that permissive and inconsistent parenting is associated with gender stereotyped misbehaviour (externalizing in boys and internalizing in girls) because children's socialized patterns of behavioural and emotional conduct go uninhibited. Stereotype incongruent misbehaviour (internalizing in boys and externalizing in girls) is associated with parental harshness that actively alters expected socialized trajectories or serves as a hostile response to unexpected misbehaviour. On the other hand, Rosenfield and colleagues [37] state that the development of psychopathology is largely a function of self-salience, which is a schema that concerns the social location of the self respective to others. It is made up of: a) an evaluation of self worth in general and compared to others, b) perceived boundaries in relationships characterized by autonomy versus connectedness, and c) the primacy and importance of an individual's own needs, interests and desires respective to that of others. People with low self-salience are predisposed towards internalizing problems because of their tendency to make negative self-evaluations and social comparisons. They also tend to blame themselves for other peoples' difficulties and internalize their stresses rather than displacing it and harming others. Individuals high in self-salience are predisposed to externalizing problems because the self is highly regarded and viewed as superior to others. Accordingly, they are unimpeded when acting against others and tend to attribute blame for personal problems to other individuals. Rosenfield and colleagues [37] cogently argue that boys are high in self-salience, while girls are low. Therefore, it is possible that boys would respond to parental risk dimensions (hostility, inconsistency, or lack of positive interaction) with externalizing behaviour whereas girls could respond with internalizing symptoms. As cited above, there is mixed support for both theories. When general trends are examined across multiple parenting dimensions [30,31] effects seem fairly consistent with the theory of self-salience. When specific patterns for certain parenting dimensions are accounted for [32], the theory of gender stereotyped misbehaviour often receives support.

Much research has suggested that boys are socialized to develop motives of personal agency and assertion whereas girls are socialized towards motives of collectiveness and collaboration [see [38], for a review]. This could account for the reported gender differences in the associations between parenting and psychopathology, but more research in this area is necessary [33]. Differences across parenting dimensions must also be considered. Despite the substantial literature base on the differential gender development patterns of males and females, we must remember that "gender" is different than biological "sex"; any given definition of gender is subjected to historical, socio-cultural, and interpretational influences [39]. As gender is a multidimensional concept [39] it is not surprising that scholars have critiqued the socially constructed gender dichotomy, highlighting the within-gender variability that is often overlooked [40,41]. Concerning the present study, limited variables in the national survey data being used has led us to examine gender as a proxy variable, where socialization is not directly measured. It is important for parenting research to account for gender in theoretical and statistical models, though the field will also benefit from literature that measures and models the mechanisms through which gender is assumed to exert influence. The present study attempts to contribute to the literature using the former approach.

### Hypotheses and Rationale

The gender-moderated relationship between parenting and psychopathology is not necessarily straightforward, where varying parenting constructs and child outcomes may alter the nature of effects. The purpose of the present investigation is twofold. First, we seek to clarify this relationship by testing the two competing theories and enriching the specificity of variables included in analysis. We will examine the gender moderated relationship between parenting and psychopathology by incorporating multiple indices of parenting (Positive Interaction, Hostile-Ineffective Parenting, and Consistent Parenting) and multiple indices of externalizing behaviour (Conduct Disorder-Physical Aggression, Indirect Aggression, Hyperactivity-Inattention) while retaining one index of internalizing psychopathology (Emotional Disorder-Anxiety).

Hypotheses have been created for each theory. When applying the *gender stereotyped misbehaviour *theory, it is predicted that parental hostility will be associated with externalizing behaviour in girls and internalizing behaviour in boys (stereotype incongruent), whereas parental inconsistency will be associated with externalizing behaviour in boys and internalizing behaviour in girls (stereotype congruent). This relationship is expected to hold with the exception of indirect aggression, an externalizing behaviour. Since overt physical aggression is discouraged amongst girls [38], it is possible that they respond to inconsistent parenting with a covert aggressive style that is more socially acceptable.

When applying the *self-salience and psychopathology *it is predicted that boys will be more vulnerable to the influences of parenting on the externalizing disorders, whereas girls will be more vulnerable to the parental influences on emotional disorder-anxiety, an internalizing disorder, regardless of the parenting construct in question. As mentioned, girls are socialized to develop schemas which emphasize the importance of others over the self creating a vulnerability to internalizing disorders. Boys are socialized to develop self-schemas that emphasize the importance of self over others predisposing them to externalizing disorders.

Both theories acknowledge that there are pervasive gender differences in the socialization experiences children encounter [38] which may translate into differential responses to parents' behaviours [5]. We will evaluate these hypotheses by examining gender moderated effects between parenting and psychopathology. Specifically, the odds ratios of interaction terms will be evaluated, indicating significant or non-significant differences in the odds of a relationship (i.e. effect size) occurring for males and females, and the direction of these differences. Our study will add to the current literature by explicitly examining gender-by-parenting interactions across several domains of psychopathology and by using an analytical approach that accounts for all child outcomes and parenting predictor variables at every cycle of measurement, adding confidence to parameter estimates. Lastly, we hope that the present investigation generates hypotheses for future research on gender differences in socialization and psychopathology. This is a very important area of research and the questions addressed in this study will not be solved by a single investigation. Also, the theories evaluated here represent only two possible explanations of the relationship between parenting, psychopathology and gender. Other factors (e.g. socialized play preferences) likely influence the development of psychopathology, as well [38].

## Methods

### Participants

Data was derived from the Canadian National Longitudinal Survey of Children and Youth (NLSCY) which was constructed to track the developmental welfare of Canadians from birth to young adulthood and study the contextual determinants of social, emotional and behavioural health [42]. Participants in the NLSCY were recruited directly from the Canadian Labour Force Survey which is the national information source for employment, allowing the generation of a representative sample of the country. This data set contains measurements collected since 1994-1995. Cycles of measurement are separated by two year intervals. Four and five year olds from the original longitudinal cohort in Cycle 1 were selected for analyses (n = 3469). Shortly after, the NLSCY chose to drop a number of siblings from data collection so that only 2 children per family contributed to the data. In Cycle 4, 1214 ten and eleven year old children remained, yielding a final weighted sample of 469,777 (50% female). The attrition rate includes siblings who were dropped and children who were lost, causing it to appear unusually high, though the overall response rate for the NLSCY is 88.54%. Data collection occurred in the survey respondents' homes with a representative from Statistics Canada. Survey respondents are the Person Most Knowledgeable (PMK) of the child which is most often the mother. This age group and timeframe were selected because the transition from early to late childhood was of particular interest. Our research group has illustrated the need for literature examining the correlates and prevalence of psychopathology in children ages 10-14, as rates in this age group may be underreported [43]. Accordingly, there is a need for investigations examining the predictors of psychopathology in the years approaching this age group. This requisite makes the developmental stage examined in the present investigation particularly relevant. More information on the NLSCY is available online, including information surrounding survey methodology, follow up rates and detailed psychometric statistics [42].

### Predictors

Scale construction and psychometric evaluation (for predictors and outcomes) was conducted by Statistics Canada prior to the data being made available to researchers [42]. Predictor variables operate at the child and proximal-family levels. The effects of age and gender were examined for each outcome variable. In the NLSCY, parenting was measured using instruments from the Parent Practices Scale [11]. Mothers responded to 17 items using a five-point Likert scale ranging from 0-never to 4-all the time. Three different parenting factors were assessed in these scales. Internal consistencies are provided in parentheses. Parental consistency (.66) assessed the degree to which parents follow through with discipline and requests. An example item is *how often does he/she get away with things that you feel should have been punished? *Positive interaction (.81) assessed parental praise and the amount of quality time spent between parents and children. An example item is *how often do you play sports, hobbies and games with him/her? *Hostile-ineffective parenting (.71) assessed annoyance, antagonism and mood-dependent behaviour expressed by parents. An example item is *how often do you get angry when you punish him/her?*

### Outcomes

The NLSCY child behaviour scales (with internal consistencies in parentheses) included physical aggression-conduct disorder (.77), indirect aggression (.78), hyperactivity-inattention (.84) and emotional disorder- anxiety (.79). On a 3 point Likert scale, PMK's responded to items such as "*How often would you say that your child kicks, bites or hits other people?*" Responses ranged from 1- never or not true to 3 - always or very true. Items for these scales were derived from the Ontario Child Health Study [44] and the Montreal Longitudinal Study [45]. Indirect aggression must be explicitly operationalized as multiple definitions exist in the literature. The NLSCY indirect aggression scale assesses the degree to which an individual uses relational strategies to inflict harm when angry at others. Consistent with precedence, the NLSCY behaviour scales were dichotomized at the 90^th ^percentile. By design, these scales are positively skewed and are intended to categorize the children who are the most dysfunctional and would likely qualify for psychiatric diagnosis [46]. The following scale scores were used to identify the 10% of children who are the most maladjusted at each of the four cycles, where individuals who scored at or above these scores fell into the top 10%: physical aggression-conduct disorder (5, 4, 4, 4), indirect aggression (3, 3, 4, 4), hyperactivity-inattention (9, 9, 9, 8) and emotional disorder-anxiety (5, 6, 7, 6).

### Analysis

Four and five year olds from the original longitudinal cohort were selected in Cycle 1 (baseline) and followed through Cycle 4. At baseline, participants who dropped out at subsequent cycles were compared to those retained at Cycle 4 to assess systematic differences between completers and non-completers. For the longitudinal analysis, the data was weighted using normalized cycle 4 longitudinal weights provided by the NLSCY. A weight is a numerical value assigned to each individual that indicates the proportion of the census-based population that the respondent represents, making parameter estimates more generalizable and robust. Normalized weights are used (i.e. an individual's weight-value divided by the mean weight for the entire sample) so that statistical tests are performed with a *N *that is the same as the sample size, as opposed to a very large *N *that would equal the number of Canadian children represented by the sample. See Statistics Canada [42] for more on weighting methodology. Each repeated response (physical aggression-conduct disorder, indirect aggression, hyperactivity-inattention, and emotional disorder-anxiety) was modeled as a function of independent variables using Generalized Estimating Equations (GEE) [47,48]. In other words, both predictor variables and outcome variables are measured at each data collection cycle and incorporated into the statistical models. There are several advantages to using GEE for studying population averaged effects of covariates on outcomes in longitudinal data structures like the NLSCY. Unlike ordinary linear regression, GEE accounts for possible correlation between repeated measurements from the same respondent at different cycles. This is accomplished by specifying a particular correlation structure that is accounted for when generating parameter estimates. We have assumed an autoregressive correlation structure (AR [1]) for the repeated responses, though other structures are possible [47]. AR [1] assumes that temporally close repeated measures will be more highly correlated than measures that are far apart in time. Regression coefficients from GEE are unbiased even if the correlation structure is misspecified [47]. The methodology is well established and the statistical literature is rich with resources relating to binary outcomes in longitudinal studies [48,49]

A main effects model (Model 1: gender, positive interaction, hostile-ineffective parenting, and parental consistency) and a model adding interactions (Model 2: all main effects *plus *gender × positive interaction, gender × hostile-ineffective parenting, gender × parental consistency) were examined. We obtained the odds ratio (OR), 95% confidence interval (CI) and associated *p-*value for each predictor in the GEE models. Statistical tests were conducted at 5% level of significance. Exploratory data analysis and GEE models were obtained from SPSS version 14 and SAS version 9, respectively.

## Results

### Analysis of Attrition

Eligible participants in Cycle 1 comprised of 3469 children. In Cycle 4, 1214 children remained, yielding a final weighted sample of 469,777 children. As mentioned, normalized weights were used in the analysis so statistical tests were performed with a normalized-weighted sample of *N = *1214. Retained participants had significantly higher scores for physical aggression, emotional disorder, and hostile-ineffective parenting than participants who were lost over the four cycles. Retained participants had significantly lower scores in positive interaction (see Table 1). An important caveat regarding the attrition rate must be mentioned. At the beginning of the NLSCY data collection, multiple siblings per family were included. Soon after, it was decided that this was infeasible and all but two siblings from each family were dropped. Thus, the true attrition rate is overstated because it includes dropped siblings (intentional) and lost participants (unintentional). We chose to include siblings in the analysis of attrition so a comparison could be made with the sample that is most representative of the Canadian population. The selective exclusion of siblings may be contributing to the fact that higher-risk participants are being retained, which runs counter to normally observed patterns in longitudinal studies. It is possible that the siblings who were dropped from the survey had systematically lower scores on psychopathology measures and hostile-ineffective parenting.

**Table 1:** Analysis of attrition comparing retained participants with complete data (n = 1214) and lost participants with complete data (n = 2255) at cycle 1 of data collection

Variable	*M(SD) *Lost	*M(SD) *Retained	F	*P*
Age of Child (y)	4.50 (0.51)	4.47 (0.47)	2.50	0.114
Physical Aggression	1.51 (1.90)	1.75 (1.96)	11.30	<0.001
Indirect Aggression	0.77 (1.42)	0.81 (1.23)	0.67	0.414
Hyperactivity	4.86 (3.47)	4.91 (3.31)	0.17	0.676
Emotional Disorder	1.97 (2.19)	2.24 (2.13)	11.34	<0 .001
Positive Interaction	14.68 (2.86)	14.40 (2.72)	7.42	0.007
Hostile-Ineffective	8.80 (3.68)	9.14 (3.62)	6.57	0.010
Parental Consistency	14.60 (3.51)	14.61 (3.32)	0.01	0.918
Income Adequacy	3.43 (1.05)	4.41 (0.93)	0.23	0.629

n = 3469; M (SD): Mean (standard deviation).

### Physical Aggression-Conduct Disorder

See Tables 2 and 3 for descriptive statistics. For model 1 (main effects only), boys had significantly higher odds of exhibiting physical aggression over time than girls. Additionally, higher scores on the hostile-ineffective parenting scale were associated with higher odds of exhibiting physical aggression over time, irrespective of gender. For Model 2 (main effects and interactions) hostile-ineffective parenting was the only covariate that remained significant and none of the parenting × gender interactions were significant. See Table 4.

**Table 2:** Descriptive statistics for categorical outcome variables at each cycle of data collection

Variable and Cycle	Females (*n *= 607)	Males (*n *= 607)	Total (n = 1214)
	
	n (%)	n (%)	n (%)
Physical Aggression 1	56 (9.2)	75 (12.4)	131 (10.8)
Physical Aggression 2	46 (7.6)	114 (18.8)	160 (13.1)
Physical Aggression 3	46 (7.6)	109 (18.0)	155 (12.8)
Physical Aggression 4	42 (6.9)	78 (12.9)	120 (9.9)
Indirect Aggression 1	77 (12.7)	45 (7.4)	122 (10.0)
Indirect Aggression 2	111 (18.3)	89 (14.7)	200 (16.5)
Indirect Aggression 3	96 (15.8)	54 (8.9)	150 (12.4)
Indirect Aggression 4	114 (18.8)	53 (8.7)	167 (13.8)
Hyperactivity 1	66 (10.9)	120 (19.8)	186 (15.3)
Hyperactivity 2	62 (10.2)	124 (20.4)	186 (15.3)
Hyperactivity 3	64 (10.5)	103 (17.0)	167 (13.8)
Hyperactivity 4	41 (6.8)	75 (12.4)	116 (9.6)
Emotional Disorder 1	82 (13.5)	90 (14.8)	172 (14.2)
Emotional Disorder 2	73 (12.0)	96 (15.8)	169 (14.0)
Emotional Disorder 3	54 (9.9)	87 (14.3)	141 (11.6)
Emotional Disorder 4	81 (13.3)	52 (8.6)	134 (11.0)

Note: presence of disorder was calculated by taking the scale score corresponding to the 90^th ^percentile for the total sample at every cycle

**Table 3:** Descriptive statistics for continuous predictor variables at each cycle of data collection

Variable and Cycle	Females (*n *= 607)	Males (*n *= 607)	Total (n = 1214)
	
	*M (SD)*	*M (SD)*	*M (SD)*
Age 1 (y)	4.49 (0.49)	4.49 (0.51)	4.49 (0.50)
Age 2 (y)	6.46 (0.49)	6.47 (0.51)	6.47 (0.50)
Age 3 (y)	8.42 (0.48)	8.42 (0.50)	8.42 (0.50)
Age 4 (y)	10.45 (0.49)	10.49 (0.51)	10.47 (0.50)
Hostile-Ineffective 1	9.14 (3.54)	9.11 (3.70)	9.13 (3.62)
Hostile-Ineffective 2	8.46 (3.36)	8.97 (3.96)	8.72 (3.68)
Hostile-Ineffective 3	8.86 (3.60)	8.86 (3.73)	8.86 (3.66)
Hostile-Ineffective 4	8.37 (3.40)	8.40 (3.92)	8.39 (3.67)
Parental Consistency 1	14.31 (3.67)	15.16 (3.17)	14.74 (3.95)
Parental Consistency 2	15.20 (3.03)	15.42 (3.13)	15.31 (3.08)
Parental Consistency 3	13.14 (2.63)	15.55 (2.98)	14.35 (3.22)
Parental Consistency 4	14.95 (3.23)	15.81 (3.02)	15.38 (3.15)
Positive Interaction 1	14.23 (2.94)	14.69 (2.89)	14.46 (2.92)
Positive Interaction 2	13.14 (2.63)	13.19 (2.51)	13.17 (2.57)
Positive Interaction 3	12.00 (2.49)	12.19 (2.51)	12.10 (2.50)
Positive Interaction 4	11.74 (2.55)	11.81 (2.59)	11.78 (2.57)

M (SD): Mean (standard deviation)

Note: Hostile-Ineffective Parenting (7 items, range 0-28), Parental Consistency (5 items, 0-20), Positive Interaction (5 items, 0-20)

**Table 4:** Multivariable results for physical aggression

	*Model 1*				*Model 2*			
		
Term	*OR*	95% CI		*p*	*OR*	95% CI		*p*
Intercept	**0.00**	0.00	0.02	<0.001	**0.00**	0.00	0.03	<0.001
Gender (Male = 1)	**1.95**	1.37	2.77	<0.001	3.10	0.24	40.68	0.388
Hostile Parenting	**1.29**	1.24	1.35	<0.001	**1.28**	1.20	1.36	<0.001
Parental Consistency	1.03	0.97	1.08	0.342	1.03	0.95	1.12	0.469
Positive Interaction	1.00	0.94	1.05	0.917	1.02	0.93	1.12	0.659
Hostile Parenting × Gender					1.02	0.94	1.12	0.624
Parental Consistency × Gender					0.99	0.89	1.09	0.801
Positive Interaction × Gender					0.96	0.86	1.07	0.467

n = 1214; OR = odds ratio; CI = confidence interval

Note: significant effects are bolded (using alpha = 0.05).

### Indirect Aggression

For model 1, boys had significantly lower odds of exhibiting indirect aggression over time compared to girls. Higher scores on the hostile-ineffective parenting scale were associated with higher odds of exhibiting indirect aggression over time, irrespective of gender. Also, higher scores on the parental consistency scale were associated with lower odds of exhibiting indirect aggression over time, irrespective of gender. In model 2, gender, hostile-ineffective parenting, and parental consistency remained significant predictors in the same direction. Additionally, higher scores on the positive interaction scale were associated lower odds of indirect aggression over time. None of the parenting × gender interactions were significant. See Table 5.

**Table 5:** Multivariable results for indirect aggression

	*Model 1*				*Model 2*			
		
Term	*OR*	95% CI		*p*	*OR*	95% CI		*p*
Intercept	**0.25**	0.07	0.97	0.046	0.68	0.13	3.56	0.646
Gender (Male = 1)	**0.50**	0.35	0.71	<0.001	**0.04**	0.00	0.46	0.010
Hostile Parenting	**1.16**	1.10	1.21	<0.001	**1.12**	1.06	1.19	<0.001
Parental Consistency	**0.94**	0.90	0.98	0.008	**0.92**	0.87	0.98	0.006
Positive Interaction	0.94	0.89	1.00	0.063	**0.91**	0.84	0.99	0.029
Hostile Parenting × Gender					1.07	0.97	1.17	0.167
Parental Consistency × Gender					1.05	0.96	1.15	0.284
Positive Interaction × Gender					1.09	0.98	1.22	0.115

n = 1214; OR = odds ratio; CI = confidence interval

Note: significant effects are bolded (using alpha = 0.05).

### Hyperactivity-Inattention

For model 1, boys had significantly higher odds of exhibiting hyperactivity-inattention over time compared to girls. Additionally, higher scores on the hostile-ineffective parenting scale were associated with higher odds of exhibiting hyperactivity-inattention over time. For Model 2, hostile-ineffective parenting was the only covariate that remained significant. None of the gender × parenting interactions reached conventional levels of statistical significance, though gender × hostile-ineffective parenting came close (*p *= .059). In this interaction, an odds ratio less than 1.00 indicates that the positive relationship between hostile-ineffective parenting and hyperactivity-inattention was weaker for boys. See Table 6.

**Table 6:** Multivariable results for hyperactivity-inattention

	*Model 1*				*Model 2*			
		
Term	*OR*	95% CI		*p*	*OR*	95% CI		*p*
Intercept	**0.02**	0.01	0.07	<0.001	**0.01**	0.00	0.07	<0.001
Gender (Male = 1)	**1.91**	1.30	2.80	0.001	6.54	0.56	77.07	0.136
Hostile Parenting	**1.26**	1.20	1.33	<0.001	**1.35**	1.24	1.46	<0.001
Parental Consistency	0.97	0.92	1.02	0.215	0.98	0.90	1.07	0.694
Positive Interaction	0.98	0.93	1.04	0.519	0.98	0.89	1.07	0.604
Hostile Parenting × Gender					0.91	0.82	1.00	0.059
Parental Consistency × Gender					0.98	0.88	1.09	0.684
Positive Interaction × Gender					1.02	0.91	1.14	0.779

n = 1214;

OR = odds ratio; CI = confidence interval

Note: significant effects are bolded (using alpha = 0.05).

### Emotional Disorder-Anxiety

For model 1, hostile-ineffective parenting was the only significant predictor, where higher scores on the hostile-ineffective parenting scale were associated with higher odds of emotional disorder-anxiety over time. In model 2, males had lower odds of exhibiting emotional disorder-anxiety over time compared to females. Higher scores on the hostile-ineffective parenting scale were associated with greater odds of emotional disorder-anxiety over time. Also, higher scores on the parental consistency and positive interaction scales were associated with lower odds of emotional disorder-anxiety over time. However, the main effects of parental consistency and positive interaction must be interpreted with caution due to the presence of significant interactions. The odds ratio for the parental consistency × gender interaction indicates that the inverse relationship between parental consistency and emotional disorder-anxiety is strongest for boys. Similarly, the inverse relationship between positive interaction and emotional disorder-anxiety is also stronger for boys. See Table 7.

**Table 7:** Multivariable results for emotional disorder-anxiety

	*Model 1*				*Model 2*			
		
Term	*OR*	95% CI		*p*	*OR*	95% CI		*p*
Intercept	**0.07**	0.02	0.26	<0.001	0.52	0.12	2.13	0.360
Gender (Male = 1)	1.00	0.67	1.50	0.993	**0.01**	0.00	0.11	<0.001
Hostile Parenting	**1.19**	1.13	1.25	<0.001	**1.15**	1.09	1.22	<0.001
Parental Consistency	0.97	0.92	1.01	0.125	**0.92**	0.87	0.98	0.005
Positive Interaction	0.96	0.91	1.02	0.161	**0.89**	0.83	0.95	0.001
Hostile Parenting × Gender					1.06	0.97	1.16	0.173
Parental Consistency × Gender					**1.11**	1.02	1.21	0.013
Positive Interaction × Gender					**1.18**	1.07	1.30	0.001

n = 1214;

OR = odds ratio; CI = confidence interval

Note: significant effects are bolded (using alpha = 0.05).

## Discussion

We hypothesized that the associations between parenting and child psychopathology would be moderated by child gender. Competing hypotheses were generated based on two theories and subsequently tested. The theory of *gender stereotyped misbehaviour *[29] led us to predict that parental hostility would be associated with externalizing behaviour in girls and internalizing behaviour in boys (stereotype incongruent), whereas parental inconsistency will be associated with externalizing behaviour in boys and internalizing behaviour in girls (stereotype congruent). This relationship was expected to hold with the exception of indirect aggression, an externalizing disorder. The second set of hypotheses, adapted from the theory of *self-salience and psychopathology *[37], led us to predict that girls would respond to all forms of negative parental experience with internalizing psychopathology, whereas boys would respond with externalization. Despite gender differences in the prevalence of certain forms of psychopathology, neither theory was supported.

### Main effects of gender and parenting

Before discussing the primary analyses, overall gender differences in prevalence will be discussed. Our findings are largely consistent with both Canadian [27,46] and American [26] prevalence estimates. Boys show higher rates of physical aggression and hyperactivity-inattention across data collection. This trend of higher rates of externalizing disorder holds with the exception of indirect aggression, where girls demonstrate higher levels at all cycles. Previous researchers have reported similar patterns [33,34]. Finally, boys and girls are similar in prevalence of emotional disorder-anxiety until the final cycle, where girls show slightly elevated prevalence at ages 10 and 11. Studies have suggested a higher vulnerability for internalization amongst boys in early childhood [50] though, similar to our findings, this appears to reverse as children approach adolescence [51]. The main effects between parenting and psychopathology are also similar to previous research [13,14,17,20]. In particular, our study further illustrates the salient influence of parental hostility and ineffective disciplinary strategies on child behavioural adjustment, as this was a significant predictor for all outcomes.

### Parent-by-gender interactions

Many studies of parenting-by-gender effects on psychopathology (ourselves included) have hypothesized interactions based on a particular intermediate mechanism - usually socialization histories or levels of a psychological construct - that varies as a function of gender. Non-replications and disagreements are likely attributable to the inconsistent ways in which these psychological markers are distributed within and between biological sexes and across samples. For example, the present study *inferred *the sex differences in self-salience, providing us with an explanation for one theory. Many studies similarly infer the mechanisms of the hypothesized interactions rather than explicitly measuring the relevant constructs. Rather than relying on inference, it seems more logical to measure the mechanism being used to explain the moderated relationship at the level of theory and using *that *as the moderator variable. We are certainly not suggesting that researchers do away with the gender variable. Rather, when possible, it makes sense to measure both gender and the explanatory mechanism if gender forms a primary component of a research question. Not only does this make theoretical sense, but it will increase statistical power by reducing the error associated with the use of binary variables such as gender. By relying on a continuous measure (e.g. a self-salience scale) that is more proximal to an outcome (e.g. child psychopathology) in a causal pathway compared to biological sex, it is possible that researchers will discover that there are much better moderators of the relationship between parenting and child psychopathology.

There were no gender-by-parenting interactions for physical aggression or indirect aggression based on the statistical models employed. Similar to previous study, the main effects models of gender, hostile-ineffective parenting, parental consistency, and positive interaction were adequate for these outcomes [14,36]. Findings for emotional disorder did not support either theory. According to the theory of gender stereotyped misbehaviour one would expect that hostility would be more important for boys, and parental consistency would be more important for girls [29]. We found no gender-by-parental hostility interaction, though there was a significant parental consistency-by-gender interaction where the effect of consistency on emotional disorder was stronger for boys. Here, we must reiterate the fact that there are both parental effects of child psychopathology and child effects on parenting, and these effects are simultaneously operative [52]. It is possible that child effects on parents also vary by a function of gender. Though not a longitudinal-causation design, Kim and colleagues [29] concluded that disturbances in emotional affectivity among girls are viewed as relatively "commonplace" and are therefore overlooked and avoided by parents. Likewise, inconsistent disciplinary patterns would be associated with patterns of child psychopathology that are more congruent with gender stereotypes. This was exhibited by the association between inconsistent parenting and poor emotional regulation amongst girls in Kim and colleagues [29]. We found that the opposite is the case. In our sample, parents of *boys *who show internalizing symptoms were more likely to be inconsistent in showing discipline. Our results add to the literature by suggesting that the effects reported in Kim and colleagues [29] are not always observed, and may differ as a function of child age. Additionally, we found that healthy interactions, not just consistency of disciplinary strategies between parents and their children contribute to emotional functioning and that this relationship is stronger for boys as well. The findings in Kim and colleagues [29] may be best suited to explain contemporaneous parental and child behaviours in early childhood, rather than persistent associations over time. Kim and colleagues [29] examined preschoolers with an average age of 4.5 years, whereas our study followed preschoolers until 10-11 years. Research has shown that the parent child associations may be complicated by third variables (such as temperament and parental self-efficacy) and that these variables may vary as a function of child-age [53]. More research in the area is needed.

These results are also incongruent with the theory of self-salience. Based on this theory, one would expect negative parental experience to elicit internalizing disorder primarily in girls and externalizing disorder primarily in boys. That is, children would respond to environmental stressors with psychological and behavioural patterns that are consistent with socialization history. This was not the case in our findings. We conclude that Rosenfield and colleagues' [37] theory is better suited to explain the main effects of gender on psychopathology, rather than the child gender-moderated associations with parenting.

The gender-by-parenting interactions modelled for hyperactivity-inattention did not reach conventional levels of statistical significance, though one came very close. Here, the effects of hostile-ineffective parenting on hyperactivity were more pronounced for girls (*p *= .059). This effect is consistent with the results of Kim and colleagues [29] who found that externalizing behaviour in girls was associated with harsh parenting. As hyperactivity and inattention are not stereotypical behaviours for girls, their manifestations may be caused or associated with harshness and overreactions from parents [29]. Our results suggest that this may be true for hyperactivity rather than physical aggression, which are both forms of externalizing behaviour. However, the results from Kim and colleagues [29] were not replicated when considering the pattern of findings from the entire analysis. Differences in research designs may account for the lack of congruity between results. Unlike Kim and colleagues [29], we did not examine maternal depression in our analysis nor did we oversample low income families. Also, there were discrepancies in measures employed. While they used general measures of internalizing and externalizing behaviour, our measures disentangled the various types of externalizing behaviour. Also, rather than employing a longitudinal statistical model, Kim and colleagues [29] examined cross-sectional correlation coefficients to examine the relationships between parental responses to child behaviour for each gender. However, as stated above, we believe the reasons for the non-replications and lack of consensus in the literature is rooted in a broader methodological error: the use of child gender as a proxy for other variables, such as socialization history.

### Study Strengths, Limitations and Future Directions

Though the tested theoretical frameworks were not supported, we feel the study still adds to the literature by illustrating the possible shortcomings of gender in understanding the relationship between parenting and psychopathology. This study relied on the use of the NLSCY, which allowed us to longitudinally examine a large number of children across the country. When formulating hypotheses, comparisons of previous research were problematic because of different developmental ages assessed between studies. Even though we did not explicitly model questions of change, our estimates reflect the general trends between 4 to 11 years of age. Our analytic approach modelled an autoregressive correlation structure, allowing us to account for serial correlations of responses over time. For significant effects, we can conclude the associations are persistent over time, between the ages of 4-5 to 10-11 years. It is also plausible that the absence of hypothesized effects in many instances is not due to low statistical power or limited sample size. Despite the measurement strengths of the NLSCY, it is limited in the included constructs and we were unable to directly assess self-salience and other possible variables of interest. The internal consistency of the parental consistency scale is somewhat low (.66) so caution should be exercised when interpreting the results from this construct. The NLSCY utilized well established parent-report measures of child psychopathology, though there are shortcomings to such methods. The mother was the respondent for the scales concerning parenting and child behaviour, leading to possible shared method variance bias. Shared method variance bias may lead to the observation of an artificial relationship, due to correlated error that is attributable to participant's response sets, rather than the meaningful correlation of true scores. Though not a focus in this study because NLSCY respondents are almost exclusively mothers, parent gender may be important to consider and innovative methods have emerged to handle such mother-partner data [54]. Socio-demographic characteristics of families were not included in the analysis. Also, the selective attrition of subjects is a challenge in all longitudinal research. Nevertheless, retained participants were actually *higher *risk than the children who were dropped or lost from data collection, suggesting that our sample does not under-represent vulnerable children. Despite the longitudinal approach, we did not disentangle the issue of directionality between child and parent constructs. Finally, we did not perform internal validation due to limited event rates. It would be helpful for future studies to perform both internal and external validation of the results.

Future parenting-by-child gender studies of child psychopathology should consider measuring the construct they believe to be responsible for the hypothesized interactions. Progress will be made by modelling this construct as the moderator variable (e.g. examining self-salience × parenting interactions) or by using path analysis or structural equation modelling to simultaneously include biological sex and the explanatory mechanism in statistical models. Researchers who focus on gender differences have indicated that biological sex is not a suitable indicator for most psychological constructs [40]. Knowing the sex of an individual tells us no more about their psychological functioning than does their shoe size. Psychosocial parenting researchers should integrate this knowledge with their research and measure *why *boys and girls respond differently to a certain risk experience, if at all. Scholars should also consider examining child-effects on parents as a function of gender or other moderator variables [53]. Heeding these suggestions may lead to a more integrated and consistent body of knowledge regarding the parental associations with psychopathology amongst boys and girls.

## Competing interests

The authors declare that they have no competing interests.

## Authors' contributions

DB conceived the study, co-wrote the application to access the national database, conducted literature review, assisted in data analysis, composed the initial draft of the manuscript and responded to reviewer revisions. AO conducted data analysis, organized results and assisted in interpretation and reporting of statistical output. CB and LT were supervisors of students DB and AO, respectively. LT planned the data analysis, wrote the analysis section and provided consultation on matters of methodology and design. CB was the literature expert, helped formulate the conceptual questions, and provided resources to access the national database. LS was responsible for cleaning data, organizing data files, conducting parts of the data analysis and providing conceptual input. All authors reviewed and edited the manuscript before submission and provided assistance with the revision process.

## Authors' information

Dillon T. Browne and Lindsay A. Smart are students at the Department of Psychology, University of Guelph, Guelph, Canada.
